# Contributions of SpoT Hydrolase, SpoT Synthetase, and RelA Synthetase to Carbon Source Diauxic Growth Transitions in *Escherichia coli*

**DOI:** 10.3389/fmicb.2018.01802

**Published:** 2018-08-03

**Authors:** Llorenç Fernández-Coll, Michael Cashel

**Affiliations:** Intramural Research Program, Eunice Kennedy Shriver National Institute of Child Health and Human Development, National Institutes of Health, Bethesda, MD, United States

**Keywords:** diauxic growth, ppGpp, acetyl-phosphate, protein acetylation, *ackA-pta*

## Abstract

During the diauxic shift, *Escherichia coli* exhausts glucose and adjusts its expression pattern to grow on a secondary carbon source. Transcriptional profiling studies of glucose–lactose diauxic transitions reveal a key role for ppGpp. The amount of ppGpp depends on RelA synthetase and the balance between a strong SpoT hydrolase and its weak synthetase. In this study, mutants are used to search for synthetase or hydrolase specific regulation. Diauxic shifts experiments were performed with strains containing SpoT hydrolase and either RelA or SpoT synthetase as the sole source of ppGpp. Here, the length of the diauxic lag times is determined by the presence of ppGpp, showing contributions of both ppGpp synthetases (RelA and SpoT) as well as its hydrolase (SpoT). A balanced ppGpp response is key for a proper adaptation during diauxic shift. The effects of one or the other ppGpp synthetase on diauxic shifts are abolished by addition of amino acids or succinate, although by different mechanisms. While amino acids control the RelA response, succinate blocks the uptake of the excreted acetate via SatP. Acetate is converted to Acetyl-CoA through the *ackA-pta* pathway, producing Ac-P as intermediate. Evidence of control of the *ackA-pta* operon as well as a correlation between ppGpp and Ac-P is shown. Finally, acetylation of proteins is shown to occur during a diauxic glucose–lactose shift.

## Introduction

Carbon catabolite repression has evolved in bacteria exposed to multiple carbon sources in order to preferentially consume a single nutrient while simultaneously inhibiting the uptake and catabolism of other, less efficient carbon sources. This phenomenon was first described by Monod () as glucose–lactose diauxic growth, which led to a paradigm shift in understanding of gene regulation (Jacob and Monod, 1961).

Currently, diauxic growth is appreciated as complex and involving several mechanisms. A major role is played by the phosphotransferase systems (PTSs). Preferential glucose uptake is accompanied by its phosphorylation to glucose-6-p, which generates PEP through the glycolytic pathway (Supplementary Figure S1). Then, PEP is the initial phosphate donor in a five step PTS phosphorelay: PEP to P∼E1 (Enzyme I) to P∼Hpr (histidine protein) to P∼EIIA^glc^ (a glucose-specific transport protein) and finally to the P∼EIIB subunit of an EIIBC membrane transport complex, which imports and phosphorylates glucose. Continued formation of glucose-6-p favors high PEP levels, which ensures that phosphate is diverted to PTS sugars such as glucose, mannose, and fructose in *Escherichia coli.* Glucose utilization is still favored over these other sugars by specific transport mechanisms (reviews see, Deutscher, 2008; Kremling et al., 2015).

Growth on glucose and the formation of glucose-6-p also favors the conversion of P∼EIIA^glc^ to EIIA^glc^. Elevated non-phosphorylated EIIA^glc^ binds to and inhibits the transport machinery for non-PTS sugars, such as lactose, maltose, glycerol, lactate, and succinate. Prevention of their uptake excludes what otherwise would constitute induction of non-PTS sugar operons and is therefore termed inducer exclusion (Deutscher, 2008). Biochemical details of EIIA^glc^ inhibition of *lacY* permease and *malK* transporter are available (Hariharan et al., 2015; Wuttge et al., 2016).

The global regulator cAMP plays an important role in diauxic shifts. In the absence of glucose, synthesis of cAMP occurs when membrane bound adenylate cyclase is activated specifically by P∼EIIA^glc^ but not by EIIA^glc^. The cAMP receptor protein (CRP) complexed with cAMP recognizes and binds to specific DNA sequences appropriately spaced in promoter regions to activate transcription in the presence of lactose or other non-PTS sugars (Popovych et al., 2009; Zhou et al., 2014). Surprisingly cellular cAMP and CRP concentrations were found to vary little during balanced growth on glucose or on lactose alone, yet during glucose–lactose diauxic lag period, cAMP increases sharply (Inada et al., 1996).

There are many additional participants. The protein Mlc represses gene expression by binding the promoter region of the target genes in absence of glucose. When glucose is present in the media, Mlc releases from its DNA binding site and binds to the dephosphorylated EIIB (Deutscher, 2008). FruR (also known as cra) is a global regulator that modulates the carbon flow in *E. coli*. FruR is a DNA binding protein that activates genes involved in transport and metabolism of glucose, while it represses expression of transporters for other sugars. Fructose 1-phosphate and fructose 1,6-diphosphate bind to FruR, which changes its conformation, reverses its binding to DNA and alters its effects on gene expression (Ramseier et al., 1995; Sarkar et al., 2008; Wei et al., 2016). Although FruR acts independently of CRP, it has been proposed to indirectly modify cAMP levels by controlling phosphorylation of EIIB (Crasnier-Mednansky et al., 1997), which suggests an intricate regulatory system.

The expression of several genes involved on the TCA cycle is also repressed by CRP during growth on excess glucose (Gosset et al., 2004) and during a glucose–lactose diauxic shift (Traxler et al., 2006). Therefore, *E. coli* growth on glucose involves a mixed acid fermentation resulting in producing acetate that will be excreted, along with other products. Recently, it has been shown that both glucose and acetate can be assimilated simultaneously under conditions of glucose excess (Enjalbert et al., 2017). When excreted acetate is taken up, it is metabolized through the *ackA-pta* pathway (Supplementary Figure S1), which produces acetyl-phosphate (Ac-P) as an intermediate metabolite. Ac-P can either acetylate or phosphorylate a variety of proteins spontaneously or enzymatically; this post-translational modification can alter catalytic activities as well as gene expression (Wolfe, 2005; Kuhn et al., 2014).

Transcriptional profiling comparisons of wild type and Δ*relA* mutants during glucose–lactose diauxic transitions reveal RelA*-*dependent changes in gene expression (Traxler et al., 2006). Altered gene expression by elevated ppGpp is not in itself surprising because there are many indications that responses to nutritional stress involve ppGpp accumulation preceded by slowing of growth and adjustments of gene expression to allow adaptations to stress (Potrykus and Cashel, 2008; Hauryliuk et al., 2015). In the transcriptomic profiling study cited above, an alternative source of ppGpp synthesis remains in the Δ*relA* mutant: the SpoT protein (Xiao et al., 1991). This is catalytically bifunctional with strong ppGpp hydrolase and a weak synthetase. Under any stress condition, the net amount of ppGpp accumulated depends on the balance between the activities of the two synthetases and single hydrolase, each of which might be regulated differently. RelA and SpoT proteins do share similar domains, but they respond to different stress situations. The RelA hydrolase domain in RelA is catalytically inactive and reason for its evolutionary persistence is puzzling. RelA is activated by sensing a deficiency of any amino acid as a limitation of codon-specified uncharged tRNA bound to RelA complexed at ribosomal A site, whose structures have been solved (Arenz et al., 2016; Brown et al., 2016; Loveland et al., 2016). There is evidence that RelA can increase the levels of ppGpp during glucose starvation – induced with α-methyl glucoside – only when amino acids are not present in the media (Gentry and Cashel, 1996). It can easily be imagined that carbon source starvation may also limit amino acids because they do have carbon skeletons formed from glycolytic intermediates and amino acids themselves can be used as carbon sources (Supplementary Figure S1).

In contrast, SpoT-dependent accumulation of ppGpp is promoted by carbon source starvation but not by amino acid starvation (Gentry and Cashel, 1996). Mechanistic clues to how carbon source exhaustion might be linked to SpoT-mediated ppGpp accumulation come from observations of Battesti and Bouveret (2006), who found regulation of the SpoT ppGpp hydrolase and synthetase is determined by acylated acyl carrier protein (Acyl-ACP). Acylation of ACP is linked to robust lipid synthesis, which in turn could be linked to acetyl-CoA accumulation. Binding of acyl-ACP to the SpoT (TGS domain) leads to conformational changes that tilt the balanced bifunctional SpoT catalytic activities to favor ppGpp hydrolysis over synthesis. Conversely, abundant uncharged ACP tilts the balance in favor of higher ppGpp synthesis and less hydrolysis. Glucose depletion also leads to altered glycolytic and/or TCA cycle intermediates that could also alter the balance of SpoT activities through other mechanisms.

We have taken a simple genetic approach as an exploratory first step toward sorting out possible mechanisms accounting for involvement of (p)ppGpp during diauxic growth transitions. The behavior of wild type cells with two (p)ppGpp synthetases is here compared to two mutant strains sharing the same (p)ppGpp hydrolase but differing in their sole source of ppGpp synthetase activity. One has only spoT synthetase and the other has only RelA synthetase. These seemingly simple comparisons have led to appreciating surprisingly complex regulatory adjustments occurring during diauxic growth shifts. A positive correlation is found between the abundance of (p)ppGpp and acetyl-phosphate (AcP) synthesized from acetate, which is secreted during minimal medium growth on glucose, and later resorbed.

## Materials and Methods

### Media and Growth Conditions

Strains and plasmids used in this study are listed in Supplementary Table S1. The different strains constructed by P1 transduction of gene deletions marked with kanamycin resistance cassette came from the Keio collection. Cells subjected to diauxic growth in 96-well plates with 300 μl of M9 minimal media containing limiting glucose (0.025%) and when indicated 0.4% lactose, 0.2% maltose, 0.4% glycerol, 0.4% lactate), 0.4% malate, 0.4% succinate, 0.2% mannose, or 0.2% fructose. Strains were grown in M9 with 0.2% glucose plates, resuspended in M9 media and diluted up to an OD_600__nm_ of 0.05 in each well. Aeration was provided by continuous shaking with temperature control in a Synergy HT plate reader (BioTek) and growth was monitored over 12 h at 37°C measuring OD_600_ at 10-min intervals. Growth under these conditions is somewhat slower than in well-aerated cultures. Minimal medium M9 liquid and plates were prepared as described in Vinella et al. (2012) and MOPS media for ^32^P labeling was prepared as described in Neidhardt et al. (1974). EMB plates were prepared as described in Barton and Melton (1987). When required, the following antibiotics were added at the indicated concentrations: 50 μg/ml ampicillin (Ap) and 20 μg/ml kanamycin (Km).

### Measurement of ppGpp Levels During Diauxic Shifts

Similar diauxic growth conditions were achieved by continuously shaking 96-wells plates in a Thermomixer (Eppendorf) and monitoring OD_600_ in unlabeled parallel cultures in the Synergy HT plate reader (BioTek). The MOPS media used for radio-labeling also contained lower phosphate (0.2 mM) to give specific activities sufficient for nucleotide labeling with 100 μCi of P^32^ in 300 μl of total volume per well. Extraction and detection of ppGpp was performed as described in Cashel (1994). Briefly, 20 μl aliquots were mixed with equal volume of 6 M formic acid, then frozen at -20°C. After two cycles of thawing and freezing, samples were centrifuged and 5 μl applied as a droplet to the surface of the TLC PEI-Cellulose F layer (Millipore), followed by immediate ascending development through wet spots by 1.5 M phosphate buffer, pH 3.4. The fully developed chromatogram was dried at room temperature and the top pH front portion containing ^32^Pi removed. Finally, autoradiographs were obtained after overnight exposure on phosphor screens, imaged with Typhoon 9400 imager (GE Healthcare/Amersham) and radioactive spots quantified with ImageJ (Schindelin et al., 2012; Schneider et al., 2012). The amounts of ppGpp and pppGpp were normalized to the sum of pppGpp + ppGpp + GTP (referred as total G) observed in the same sample.

### Measurements of ppGpp Decay

Cells were grown in MOPS media contained 0.2 mM phosphate labeled with 100 μCi of P^32^ as above. After 1 h of incubation at 37°C, amino acid starvation was induced by adding 1 mg/ml SHX for 30 min and then 200 μg/ml of chloramphenicol was added. Samples were taken every 30 s. Extraction and detection of ppGpp was performed as described above. The amounts of ppGpp was normalized to total G observed in the same sample and plotted on a semilogarithmic scale.

### Measurement of Acetate Levels

Cells were grown in minimal media with 0.2% glucose to an OD_600_ of 0.2, collecting 5 ml samples during growth. Each sample was centrifuged, and the supernatant filtered with a 0.22 μm filter (Millipore). The Acetate Colorimetric Assay from Sigma-Aldrich (cat. # MAK086-1KT) was used to measure acetate levels in the culture supernatant using acetate standards from 1 to 10 nmole/well; if necessary, samples were diluted to be in the range of the standard curve. The amounts of acetate are normalized to OD_600_.

### Measurement of Acetyl-Phosphate (AcP) Levels

Levels of Ac-P were measured by a coupled assay in which added ACP kinase quantitatively converts AC-P to acetate and ATP, which is detected by luminescence (Weinert et al., 2013). Briefly, cells were grown to an OD_600_ = 0.5, spun and resuspended at 1 OD_600_/50 μl in ice cold assay buffer (10 mM NaPO_4_ pH 7.5, 10 mM MgCl_2_, and 1 mM EDTA). After setting aside an aliquot for measuring protein content, the cells were lysed in 1% SDS 95°C for 5 min and Ac-P was extracted with 3 M HClO_4_, neutralized with saturated KHCO_3_ and cellular ATP removed from the lysate with activated charcoal. Then Ac-P was converted to acetate and ATP using acetate kinase (AckA) and ATP content determined in triplicate using CellTiter-Glo Luminescent Cell Viability Assay (Promega, cat. # G7570). Luminescence detection with a Synergy HT plate reader was quantitated with a 0.1 to 10 mM ATP standard curve. The amount of ATP was normalized to protein content determined by nanodrop spectra and expressed as a fraction of wt strain values.

### Gel Electrophoresis and Western Blotting

Cells from 1 ml culture were centrifuged and 1X LDS sample buffer with reducing agent (Thermo Fisher) was added to the cell pellet, sufficient to give an OD_600_ of 2.5. Samples were boiled and resolved on a NuPAGE 10% Bis-Tris Gel in MOPS SDS Running Buffer from Thermo Fisher. Proteins were transferred onto a nitrocellulose membrane with an iBlot gel transfer system. Lysine-acetylated proteins were detected with mouse anti-AcK antibody (Cell Signaling Technology, cat. # 9681S) diluted 1/1000 in TBS-T with 5% of BSA, incubated overnight at 4°C and developed with peroxidase conjugated anti-mouse antibody. The signal was detected with a chemiluminescent reaction, using ECL^TM^ Western Blotting kit from GE Healthcare, and detected by a BioRad Molecular Imager ChemiDoc XRS System.

### Reverse Transcription-Quantitative Polymerase Chain Reaction

Expression of *pta* and *ackA* was determined by reverse transcription-quantitative polymerase chain reaction (RT-qPCR). Briefly, two independent cultures for each strain were grown in M9 minimal media with 0.025% glucose and 0.4% lactose at 37°C. Samples were taken during the diauxic growth and RNA isolated with Trizol (Thermo Fisher, cat. # 15596026) as indicated by the manufacturer. The RNA samples were retrotranscribed into cDNA using the High Capacity cDNA Reverse Transcription kit from Applied Biosystems and target genes were amplified with SYBR Green PCR Master Mix from Applied Biosystems in a LightCycler 480 instrument. The relative gene expression was determined with the comparative CT method, also known as 2^-ΔΔC_t_^ (Schmittgen and Livak, 2008) with three technical replicates (six values for each experimental condition). The *parC* gene was used as endogenous control. Primers used in this study are listed in Supplementary Table S2.

## Results

### Diauxic Lag Times

**FIGURE 1:**
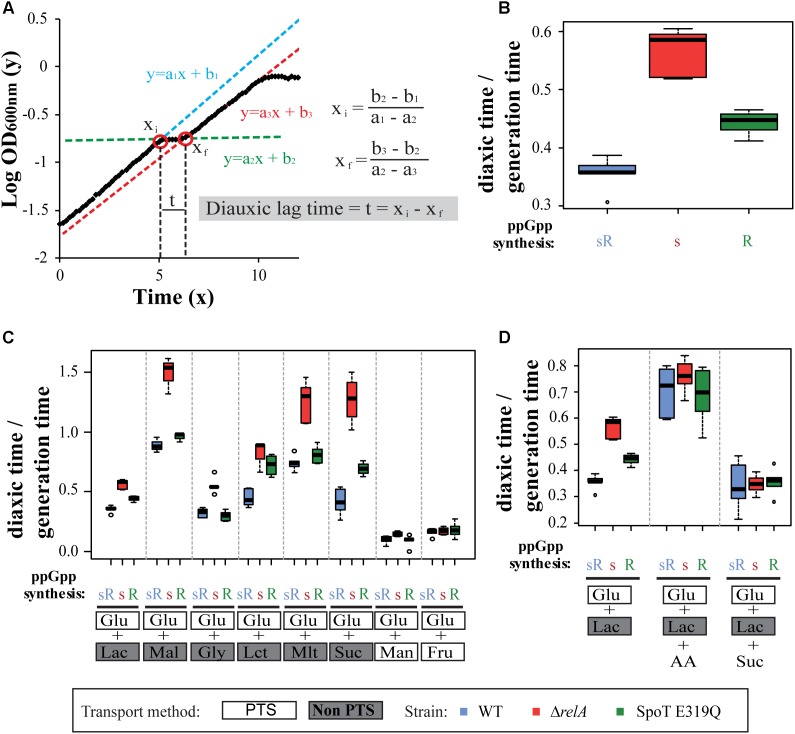
Effect of RelA and SpoT synthetase on the diauxic shift. **(A)** Representation of the diauxic growth as functions of intersecting lines. **(B)** The ppGpp synthetase test strain set: MG1655 (wt), Δ*relA*, and SpoT E319Q were grown in M9 with 0.025% glucose and 0.4% lactose for 12 h and OD_600__nm_ measured every 10 min. Cell types indicate (ppGpp synthetases present: s = SpoT synthetase; R = RelA synthetase): sR = wild type; s = Δ*relA, spoT*+; R = *relA+* spoT E319Q synthetase defective mutant. **(C)** The same strain set as in **(B)** are grown in M9 with 0.025% glucose but with different secondary sugars: 0.4% lactose (Lac), 0.2% maltose (Mal), 0.4% glycerol (Gly), 0.4% lactate (Lct), 0.4% malate (Mlt), 0.4% succinate (Suc), 0.2% mannose (Man), and 0.2% fructose (Fru). **(D)** As **(B)** grown in M9 with 0.025% glucose and 0.4% of lactose and additionally adding (0.4%) succinate or 20 amino acids (each 50 μg/ml) (AA). Ratios of diauxic time normalized to generation times of three independent experiments with duplicate wells (six values) were plotted as box plots. Bottom and top of the colored box represent first and third quartiles, and the band inside the box is the median. Whiskers represent the minimum and maximum data, while circles are outliers (single points). PTS and non-PTS sugars are indicated.

The diauxic lag time is defined here as the time required for *E. coli* to adapt from growing in a PTS sugar (glucose) to a non-PTS sugar (i.e., lactose). As shown in **Figure 1A**, diauxic growth on glucose and lactose has a typical shaped curve: cells grow exponentially on glucose until it is exhausted (line 1, blue), then cells stop growing (line 2, green) and adjust gene expression that allow resumption of growth on lactose (line 3, red). The diauxic lag time is extrapolated as the time (t) between two straight line intersects (Xi and Xf) on a logarithmic plot of bacterial growth (OD_600__nm_) versus time; see **Figure 1A**.

It might be expected that initially fast-growing cells will have a shorter diauxic lag time than slow-growing cells; therefore, we normalized the diauxic lag time to the generation time during first sugar (glucose) growth. This simple mathematical approximation does allow quantitative comparisons of diauxic lag times for different strains. Similar to the method described by Monod (1949) to measure lag phase, our method takes into consideration the retardation and acceleration phase produced before and after the diauxic lag time respectively.

### ppGpp Synthetase-Specific Effects on Diauxic Lag Times

We assess the role of each synthetase on diauxic lag times by comparing wild type (wt) and two mutants expressing only RelA or SpoT synthetase activity. For these comparisons to be meaningful, each missing synthetase mutant should be a near null allele and the hydrolase activities shared by all three strains should be demonstrably equivalent.

The RelA synthetase mutant used is a large ORF deletion. Because a RelA+ Δ*spoT* is not viable (Xiao et al., 1991), a point mutation in SpoT synthetase was used. We are assured that the missense SpoT E319Q mutant (Harinarayanan et al., 2008) lacks significant synthetase activity, because Δ*relA spoT* E319Q double mutant does not grow on minimal glucose and therefore is a phencopy of a ppGpp^0^ strain (Supplementary Figure S2A). Supplementary Figure S2B shows the SpoT E319Q *relA*+ synthetase and a wt strain accumulate (p)ppGpp similarly in response to serine hydroxamate, indicating the strong RelA synthetase is unaffected by the SpoT synthetase mutant. Finally, comparison of the wt cellular decay rates of radio-labeled ppGpp in the SpoT E319Q mutant reveals equivalent rates of decay (Supplementary Figure S2C). We take this as evidence that the *spoT E319Q* mutant is both severely deficient in synthetase and this deficiency does not appreciably alter the hydrolase activity encoded at the neighboring domain.

Here, three strains, MG1655 (wt), CF18005 (Δ*relA*), and CF18011 (SpoT E319Q) are used repeatedly as a test set during this study. The experiments are carried out in microtiter dishes in three independent experiments, each with duplicate wells, which yields six lag time values statistically represented as boxplots. Average and standard deviation of lag times and growth rates for every growth condition can be found in Supplementary Table S3. **Figure 1B** shows the glucose–lactose diauxic shift for the strain set under our conditions: the wt (sR, blue) with both synthetases is compared to either Δ*relA* strain expressing only the SpoT synthetase (s, red) or to the SpoT E319Q strain expressing only the RelA synthetase (R, green). A hierarchy of lag times for the classical glucose–lactose diauxic shift is observed (**Figure 1B**) with wt the quickest, the s strain the slowest and an intermediate time is seen for the R strain. These findings for the wt (sR) and Δ*relA* mutant (s) strain agree with changes in gene expression and diauxic lag times observed in transcriptomic studies (Traxler et al., 2006).

Our data show that ppGpp controls the length of diauxic lag. The hierarchy observed between strains is taken to mean that each ppGpp synthetase contributes to the adjustments determining diauxic lag times but to different degrees. There is also the widely accepted notion that nutritional stress increases ppGpp to facilitate adaptive gene expression to ameliorate the stress. If valid, this predicts the more ppGpp during the shift, the faster the adjustments, the shorter the lag time.

**Figure 1C** shows diauxic growth shift responses of the strain set from glucose to five non-PTS carbohydrates other than lactose: maltose, glycerol, lactate, malate, or succinate. It can be seen that in each case the diauxic lag times vary widely but absence of *relA* synthetase always is the most effective for prolonging the lag time relative to two strains with RelA synthetase. The relative intermediate hierarchical position of the SpoT mutant varies markedly among the shifts presented. It may be the same as wt (glycerol), nearly the same (maltose, malate), in the middle (lactose, succinate), or near the slowest strain (lactate). The reason for this hierarchy is unknown; it might suggest altered regulatory responses to ppGpp or different effects on ppGpp synthetase activities in response to different carbon sources.

**Figure 1C** also shows control shift experiments from glucose to two other PTS sugars: fructose and mannose. Both shifts result in the shortest diauxic lag times for all members of the test set of strains and abolition of their hierarchical differences. The discriminating effects of ppGpp synthetases seem to apply to shifts to non-PTS sugars.

### Effects of Adding Amino Acids on Diauxic Shifts

As mentioned before, RelA synthetase activation can be stimulated by glucose starvation only during amino acid limitation (Gentry and Cashel, 1996). Considering that the observed prolongation of diauxic lag times seems longest when RelA is missing, it is plausible that diauxic shifts involve amino acid starvation. These shifts in minimal media lead to exhaustion of available glucose as both an energy source and anabolic substrates. Since time is required before alternative carbon sources provide these functions, starvation for amino acids or their tRNA charging can be expected. One test is simply to add a mixture of all 20 amino acids (50 μg/ml each). **Figure 1D** shows that adding the amino acid mixture during a glucose–lactose shift does abolish the hierarchy. It can be predicted that catabolism of some amino acids in the mixture might provide transient sources of alternative carbon sources or of TCA intermediates during glucose exhaustion.

This notion is reinforced by the parallel observation (**Figure 1D**) that adding succinate during the glucose–lactose shift has a similar effect as adding the amino acid mixture, it also abolishes the test set lag time hierarchy. Analogous experiments adding malate during a glucose–lactose shift do not mimic the addition of succinate (data not shown).

### The ppGpp Effect on Diauxic Lag Time Does Not Involve Classic Regulators

**FIGURE 2:**
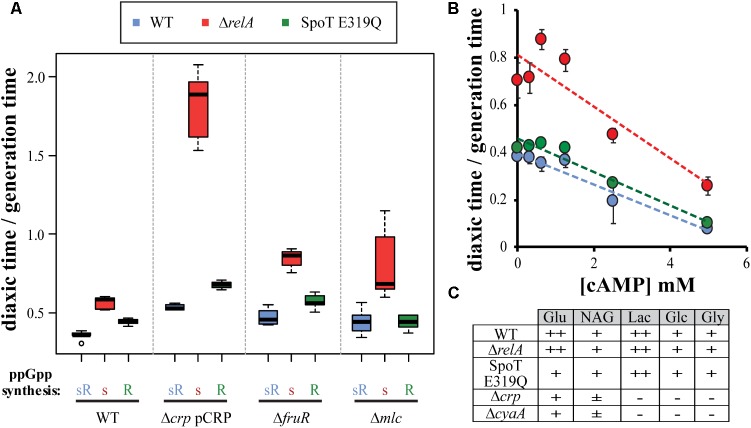
CRP-cAMP, Mlc, and FruR do not alter diauxic shift effects of ppGpp mutants. **(A)** *crp* pHA7 (pCRP), Δ*fruR*, and Δ*mlc* were introduced into the test strains set and subjected to glucose–lactose growth shifts. **(B)** Wt, Δ*relA*, and SpoT E319Q mutant strains were subjected to glucose–lactose shifts with increasing amounts of cAMP (0 to 5 mM) added to the growth media. **(C)** Use of EMB plates to assess the ability of wt, Δ*relA* and SpoT E319Q mutant strains to utilize glucose (Glu), *N*-acetylglucosamine (NAG), lactose (Lac), gluconate (Glc), and glycerol (Gly) as compared to Δ*crp* and Δ*cyaA* mutants.

As mentioned earlier, diauxic shift behavior is regulated by additional transcription factors such as Mlc, FruR or CRP-cAMP (Deutscher, 2008; Sarkar et al., 2008), that could be regulatory targets of ppGpp. To asses if the ppGpp effect is dependent or not of the classic regulators, different mutants were used. Mlc and FruR deletion mutants can traverse diauxic shifts, allowing transducing these deletions into the test set of strains to measure glucose–lactose growth transitions. The typical hierarchies were found unaffected (**Figure 2A**). Deletions of *crp* or *cyaA* cannot grow in lactose but they can grow when CRP is constitutively expressed from the plasmid pHA7 (Aiba et al., 1982). It has been shown that ppGpp affects the transcriptional initiation of one of the *crp* promoters (Johansson et al., 2000). If ppGpp alters diauxic shift through controlling *crp* expression, the presence of pHA7 might alter the hierarchy. The Δ*crp* pCRP *relA* mutant strain does show a lag time that is longer than even the same *crp*+ strain for glucose–maltose shifts, which was the most prolonged lag time so far observed (**Figure 1C**). Evidently there is a CRP effect, but it does not alter the hierarchy. Since ppGpp might still alter cAMP levels, possible effects of titrating additions of cAMP to growth media up to 5 mM on glucose–lactose diauxic lag times were examined with the test set of strains (**Figure 2B**). An inverse linear relationship is observed between the amount of cAMP added and the lag time, but the typical hierarchy again persists. Finally, the ability to utilize different sugars was estimated with EMB plates (Barton and Melton, 1987) with the test set of strains comparing effects of *crp* or *cyaA* mutants (**Figure 2C**). The test set of strains are found to utilize all five sugars examined, while *crp* or *cyaA* mutants can use only glucose and partially utilize *N*-acetyl glucosamine. In summary it is concluded that diauxic shift differences are not likely to be explained in terms of Mlc, FrurR, or CRP-cAMP effects.

### Measurements of (p)ppGpp Levels During a Glucose to Lactose Diauxic Shift

**FIGURE 3:**
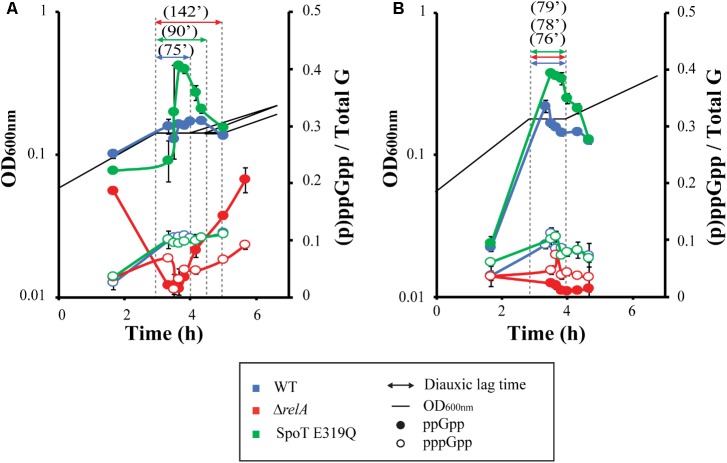
(p)ppGpp levels during diauxic shifts. MG1655 (wt), Δ*relA*, and SpoT E319Q strains were grown and labeled with P^32^ in MOPS media with 0.025% glucose and 0.4% lactose in absence **(A)** or presence of (0.4%) succinate **(B)**. Black lines indicate OD_600__nm_ of the different strains. Dashed lines determine the limit of the diauxic shift, with the time of each shift indicated by double arrows. Full symbols represent measurement of ppGpp, while open symbols represent pppGpp.

The observations described above emphasize the need for a quantitative survey of the levels of (p)ppGpp during glucose–lactose shifts (**Figure 3A**).

The first samples taken represent exponential growth on glucose, about an hour before growth arrest. These reveal (p)ppGpp basal levels are similarly elevated for all three strains. The observed pre-lag basal levels do reflect the now familiar hierarchy. The guanosine pentaphosphate behaves differently with basal pppGpp levels for the strain set; all very low and almost identical.

The next samples reflect effects during the diauxic lag. The ppGpp levels for the wild type strain increase about 30% and plateau after the quick rise and then begin to drop back to near basal levels when growth resumes. For the SpoT E319Q mutant with (R), the increase of ppGpp is more marked, with a rapid rise that peaks in mid-lag, rapidly falling, then slowing during resumption of growth. A similar observation was made in Gentry and Cashel (1996), with a RelA+ strain containing a truncated SpoT protein without synthetase activity during glucose exhaustion; higher levels of ppGpp were found compared to full length SpoT. For the Δ*relA* strain ppGpp drops precipitously from pre-lag basal levels to quickly become barely detectable early in the lag period; it then slowly climbs to reach pre-lag basal levels after the lag. The dramatic drop for the Δ*relA* mutant seen here is reminiscent of observations during an amino acid starvation of a Δ*relA* mutant (Lagosky and Chang, 1981). The ppGpp content of strains with and without RelA are almost mirror images of one another. As for pppGpp, both strains with RelA show a rapid fivefold increase in pppGpp at the onset of the lag followed by a plateau that persists even when growth resumes. The pppGpp of the Δ*relA* strain drops quickly to barely detectable, then slowly climbs during and after the lag to growth with higher basal levels than during pre-lag.

Overall (p)ppGpp accumulation behavior during the diauxic shift is consistent with the notion that the more ppGpp, the shorter the lag. There is a possible proviso that the spike of ppGpp to levels higher than wt for the R strain, coupled with a slower lag time could perhaps be explained by the inhibitory effects of ppGpp on growth (Mechold et al., 2013). More thorough kinetic sampling is required to learn whether the precipitous ppGpp drop in the Δ*relA* strain during the lag is due to restricted synthesis, activated hydrolysis or both.

### Measurements of (p)ppGpp Levels During the Standard Shift but With Succinate Present

Adding succinate eliminates the hierarchy between our strain set (**Figure 1D**). We hypothesize that it may affect the (p)ppGpp accumulation pattern observed in **Figure 3A**. Therefore, we surveyed the levels of (p)ppGpp during a glucose–lactose shift in presence of succinate (**Figure 3B**). The first thing noticed is that succinate speeds growth on glucose (0.46 dbl/h glucose–lactose vs. 0.66 dbl/h glucose-lactose-succinate) and predictably lowers basal levels of (p)ppGpp (Potrykus et al., 2011; Mechold et al., 2013). During the lag periods of both RelA+ strains (wt and SpoT E319Q), the levels of ppGpp and pppGpp increase from these lower basal levels to levels similar to those found in the absence of succinate. Thus, except for lowered basal levels, the ppGpp content of both RelA+ strains mirror changes seen without succinate. For the Δ*relA* strain, the already low levels of ppGpp drop even lower and fails to increase, as occurs when succinate is absent, as if succinate or its metabolites might inhibit SpoT synthetase or stimulate SpoT hydrolase. The pppGpp levels of the three strains behave similarly as without succinate (**Figure 2A**), except for the Δ*relA* strain that remains unchanged and almost undetectable during diauxic shift.

The most striking differences occasioned by the presence of succinate for all three test strains is the short lag periods and elimination of the hierarchies regardless of their patterns of (p)ppGpp accumulation. This effect of added succinate clearly contradicts the notion that the presence of (p)ppGpp necessarily facilitates adjustments during the diauxic shift. Instead when RelA is deleted, and (p)ppGpp levels are very low, the lag times are as fast as when ppGpp spikes to higher levels. This raises the question as to whether the effect of succinate is able to overcome the effect of ppGpp over diauxic shift.

### Succinate Controls the Uptake of Acetate

**FIGURE 4:**
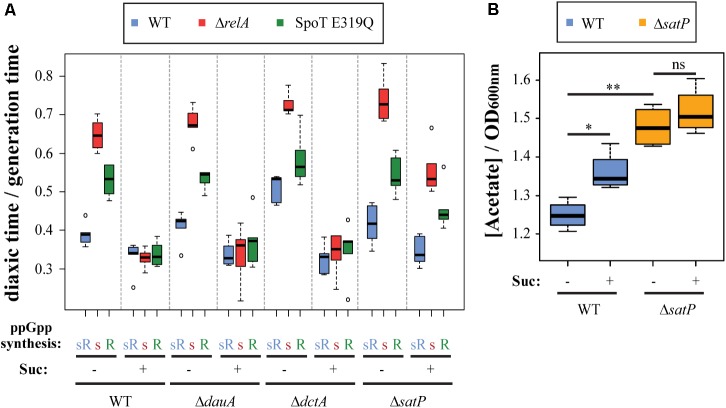
Succinate transport blocks acetate uptake, inhibiting ppGpp effects on diauxic shift. **(A)** Effects of deletion of succinate transporters DauA, DctA, or SatP on the ability of succinate to abolish ppGpp synthetase-specific effects on diauxic lag times. See Supplementary Figure S1 for succinate and acetate transport. **(B)** Concentration of acetate in the media, expressed in mM (nmole/μl) per unit of OD_600__nm_, after growing MG1655 (wt) and Δ*satP* mutant in M9 with glucose in presence or absence of succinate. Significant differences were indicated: ^∗^*p* < 0.05; ^∗∗^*p* < 0.001; ns, no significant.

A key question that now emerges (**Figures 1D**, **3B**) is how succinate addition alters the roles of ppGpp in diauxic shifts independent of high or low ppGpp levels. Succinate must be transported into cells to have intracellular cellular effects. Succinate transport mechanisms are redundant and controlled by DctA, whose expression requires CRP (Lo, 1977; Gosset et al., 2004), by DauA (Karinou et al., 2013), and by SatP (Sá-Pessoa et al., 2013). Therefore, glucose–lactose diauxic shift transitions of our strain set were monitored in presence or absence of succinate comparing effects of deleting each of the three succinate transporters (**Figure 4A**). In the absence of succinate, deletions of each of the three transporters does not alter the usual lag time hierarchies. When succinate is present similar responses as wt are seen only for Δ*dauA* or Δ*dctA* single deletion mutants. Presumably, the uptake of succinate persists due to residual redundant activities of non-mutant transporters in these strains. The Δ*satP* deletion behaves differently. It uniquely fails to abolish the diauxic lag hierarchy in presence of succinate despite the presence of active *dauA* and *dctA* sources of continued succinate uptake. Clearly succinate alone is not disrupting the hierarchy. The difference between the three succinate uptake systems is that SatP can also transport acetate as well as succinate (see Supplementary Figure S1), but succinate is a competitive inhibitor of acetate transport by SatP (Sá-Pessoa et al., 2013). A second source of acetate uptake is ActP, but it requires CRP to be expressed during the diauxic shift (Traxler et al., 2006), due its co-transcription with *acs* (Gimenez et al., 2003).

Secretion of extracellular acetate during growth on glucose arises from acetate overflow. It is important to highlight that most of the studies that show acetate overflow (Wolfe, 2005; Sá-Pessoa et al., 2013; Enjalbert et al., 2017), use higher amounts of glucose than what is used in this study (0.025%). To partially answer this, the levels of acetate secreted in the media were measured in presence or absence of the SatP transporter during growth in M9 minimal media with 0.2% of glucose to an A600 of 0.2 in presence or absence of succinate (**Figure 4B**). The presence of succinate significantly elevates media acetate levels for the wt strain, consistent with succinate impairment of SatP-mediated import (Sá-Pessoa et al., 2013). Media acetate levels are further elevated in the *satP* deleted strain but to the same extent in the presence or absence of succinate; this seems to indicate the effect of succinate on acetate levels specifically acts through SatP import and not elsewhere. Nevertheless, acetate levels found in media levels are not governed exclusively by uptake but also by the balance of rates of secretion, uptake and utilization. The *ackA-pta* pathway is bidirectional; it can be involved in both secretion and utilization of acetate (Enjalbert et al., 2017). We next asked if disrupting this pathway can alter media acetate level and the diauxic lag hierarchy found in the ppGpp synthetase strain test set.

Under the same growth conditions as in **Figure 4B**, the wt levels of acetate in the media (1.25 mM ± 0.04/OD_600__nm_) drop to 0.45 ± 0.07 mM acetate/OD_600__nm_ when *ackA* is deleted. Similar results were obtained in absence of *pta* in M9 with 2% glucose (Chang et al., 1999), where a reduction of acetate levels was observed. Instead, they show an increase in other secreted compounds such as pyruvate or lactate, suggesting a redirection of the metabolism when *ackA-pta* pathway is blocked.

### The Effect of ppGpp Over Diauxic Growth Is Related to Acetyl-Phosphate

The above information leads us to hypothesize that succinate blocks the SatP-mediated uptake of the acetate secreted before glucose exhaustion and abolishes diauxic lag differences arising from different synthetic sources of ppGpp. It is important to mention that the bidirectional *ackA-pta* pathway (Supplementary Figure S1) produces the high energy acetyl-phosphate (Ac-P) as an intermediary metabolite that can post-translationally modify many proteins, sometimes accompanied by functional consequences. This occurs by either acetylation or phosphorylation that can occur spontaneously or enzymatically (Wolfe, 2005; Kuhn et al., 2014).

**FIGURE 5:**
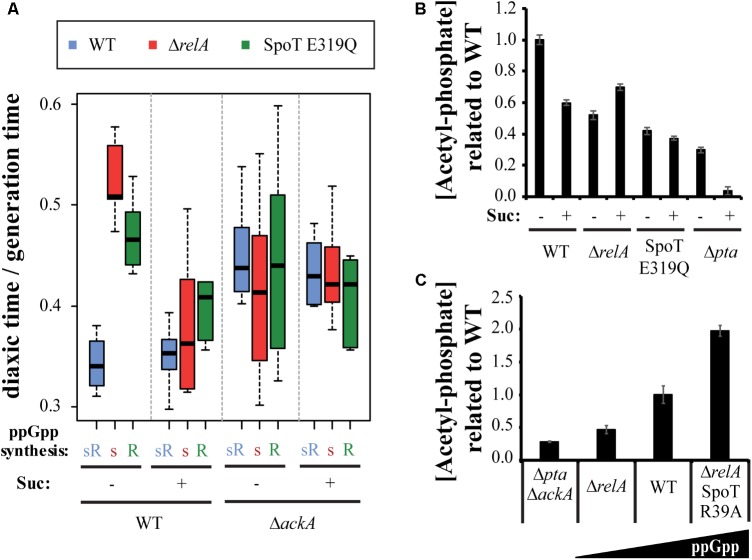
ppGpp correlates with acetyl-phosphate levels. **(A)** The effect of deleting *ackA* with or without succinate on diauxic lag times of the of wt, Δ*relA*, and SpoT E319Q mutant strain set. **(B)** Levels of Ac-P are altered in the mutant strain set grown on M9 + glucose with or without succinate and the effect of a *pta* deletion. **(C)** Levels of Ac-P in strains grown in M9 + glucose containing different basal levels of ppGpp.

During growth on glucose, Ac-P can be synthesized from acetyl-CoA by Pta or from acetate by AckA and *vice versa*. As mentioned above, there is a balance between excretion and uptake of acetate, that will result in balanced levels of Ac-P inside the cell. During glucose exhaustion we hypothesize that this balance will be shifted to favor uptake of acetate as the predominate source of Ac-P catalyzed by AckA. If succinate indeed affects diauxic shifts by blocking the entrance of acetate resulting in decreased Ac-P; this hypothesis can be tested by deleting *ackA*, which would produce a similar effect but at the level of converting acetate to Ac-P rather than blocking uptake of acetate. If the succinate effect is due any other reaction than phosphorylation of acetate to Ac-P, then deleting *ackA* would have no effect. The diauxic shift of the three test strains was determined in wt and Δ*ackA* backgrounds, with and without succinate (**Figure 5A**). In the Δ*ackA* background the hierarchy disappears regardless of added succinate, in support of the hypothesis. Since the *ackA* deletion prevents the formation of Ac-P from acetate, the implication is that ppGpp somehow affects this step.

A related possibility is that measured levels of Ac-P might be different among the set of three strains growing, not in the minimal media during diauxic shifts, but growing exponentially in M9 minimal medium with 0.2% dextrose ± succinate. **Figure 5B** reveals that compared to wt, Ac-P levels in the absence of either the *relA* or *spoT* synthetase are low (twofold), as if the levels of Ac-P are lower than wt before the diauxic lag begins. The presence of succinate can be seen to reduce the amount of Ac-P in a wt strain. As mentioned above, during growth on glucose Ac-P can alternatively be produced by Pta from acetyl-CoA as well as by AckA from acetate. The succinate-dependent drop in Ac-P can be assigned to the blockage of acetate uptake. The Δ*pta* strain might be expected to allow estimates of each contribution because it should amplify Ac-P formed from acetate as well as to eliminate Ac-P formed from acetyl-CoA. **Figure 5B** shows that without added succinate the levels of Ac-P are strongly reduced relative to wt but drop further to almost 0 when succinate is added.

### ppGpp Controls the Levels of Acetyl-Phosphate

We deduce a regulatory relationship exists between Ac-P and ppGpp because the effects of ppGpp on diauxic shifts seem to depend on the presence of Ac-P (**Figure 5A**) and because the absence of either the RelA or SpoT synthetase is associated with reduced assayed levels of Ac-P (**Figure 5B**). Indeed, assays of Ac-P basal levels reveal an approximate correlation with increased ppGpp basal levels in ppGpp basal level mutants shown schematically in **Figure 5C**. Compared to a Δ*ackA*Δ*pta* double mutant, the Ac-P basal levels range from a significant increase in a Δ*relA* strain, to higher in wt and even higher in a Δ*relA spoT* R39A mutant, whose ppGpp basal levels are eightfold higher than wt (Sarubbi et al., 1988). The Ac-P levels detected in the Δ*ackA*Δ*pta* double mutant are a bit higher than expected, but it could be due basal noise levels.

Regulation of Ac-P by ppGpp could be due to effects on the *ackA-pta* pathway. The *ackA* and *pta* genes form an operon (Supplementary Figure S3A) with two reported transcripts: one for only *pta* and the other for both genes (Kakuda et al., 1994). Each transcript was measured by RT-PCR during the diauxic shift in presence or absence of RelA and it was found that relative to wt, transcription of *ackA* and *pta* (Supplementary Figures S3B,C) are clearly reduced during the diauxic shift in absence of RelA. Both expression patterns are identical, suggesting that these potential effects of ppGpp are exerted over the whole *ackA-pta* operon. High basal levels of ppGpp during growth on glucose prior to glucose exhaustion (**Figure 3A**) imply that ppGpp might activate *ackA-pta* transcription during this growth period as well as during the lag period for *relA+* strains but not for the *relA* mutant with low ppGpp and longer diauxic lag times. The promoters for this operon are fairly well-defined by transcript size, start sites from RNA-seq data and -10 sequence promoter predictions (Kakuda et al., 1994; Münch et al., 2005; Salgado et al., 2013). Two possible promoters are predicted upstream of *ackA-pta* operon (Supplementary Figure S3A). Both have AT-rich discriminator sequences between transcript -10 and +1 region, suggesting they might be activated by ppGpp (Travers, 1980; Da Costa and Artz, 1997; Gummesson et al., 2013). This behavior is consistent with a possibility of a direct stimulatory regulatory effect of ppGpp on transcription on the *ackA-pta* operon which might lead to increased acetylation of proteins during diauxic lag time.

**FIGURE 6:**
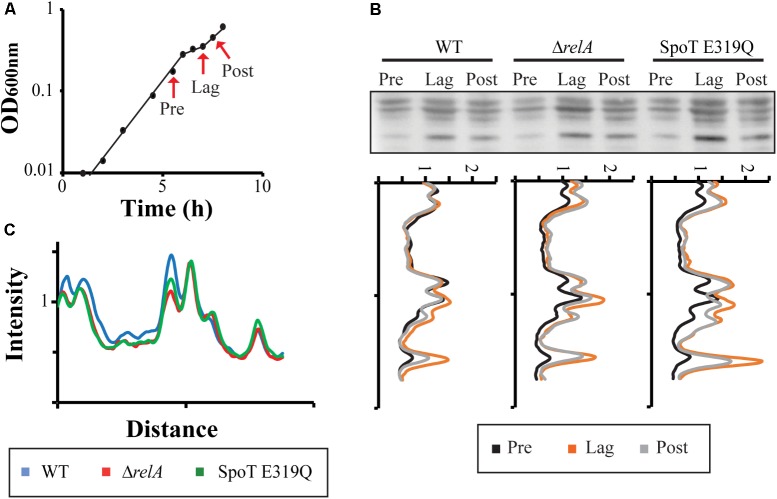
Acetylation of proteins during diauxic shift. **(A)** Sample times (Pre = before diauxic shift, Lag = during diauxic shift, Post = after diauxic shift) taken during the glucose to lactose diauxic shift. **(B)** Western blots with monoclonal antibody against acetylated lysine at sample times for the strain set: wt, Δ*relA*, and SpoT E319Q. The intensity profile of each strain comparing the amount of acetylated lysine proteins at different time points is shown. Full Western Blot is shown in Supplementary Figure S4. **(C)** The intensity profile of acetylated lysine proteins at the “Pre” sample point is compared between the different strains.

Several proteins involved in glycolysis or the TCA cycle are known to be acetylated by Ac-P (Kuhn et al., 2014; Venkat et al., 2017). However, these observations were made under conditions that optimize Ac-P accumulation rather than the more standard growth conditions used here. The growth conditions of our diauxic growth experiments are not likely to result in maximal Ac-P abundance. Nevertheless, Ac-P levels in our experiments can be correlated with even low basal ppGpp levels and Ac-P accumulation seems related to ppGpp effects on diauxic lag times. It is therefore important to ask if acetylation of proteins actually occurs during our diauxic shift conditions and if so, whether protein acetylation differs among our standard set of three strains. In **Figure 6A**, samples were taken at different times to detect acetylated lysine by Western blots: (Pre) during exponential growth on glucose before the diauxic shift; (Lag) during the diauxic transition lag time; and (Post) during exponential growth on lactose after the diauxic lag. The quantitative tracings reveal the highest abundance of acetylated proteins does occur during the diauxic lag and these levels diminish after the diauxic shift (**Figure 6B**). Higher levels of acetylation occur for the SpoT E319Q mutant with active RelA synthetase during the diauxic shift than seen in the wt strain. This is consistent with the time when higher levels of ppGpp are detected (**Figure 3A**). Comparing the amount of acetylated proteins during growth in glucose before the diauxic shift (**Figure 6C**, “Pre” sample) reveals a reduction on acetylated proteins in both Δ*relA* or SpoT E319Q strains. This is consistent with lower amounts of Ac-P (**Figure 5B**) and lower levels of ppGpp (**Figure 3A**) at this time. No differences were detected in Coomassie stained gels (data not shown).

## Discussion

A very early comparative study of ppGpp accumulation responses to starvation for amino acids, glucose and even a glucose–succinate transition gave rise to twin notions that RelA is needed to quickly activate ppGpp synthesis (Lazzarini et al., 1971). When *relA* was deleted, the amino acid starvation response was abolished and transient slow accumulation of ppGpp, but not pppGpp, was seen during the diauxic lag period. It was thought then that the *spoT1* mutant strain used in these experiments was defective only in hydrolysis and not yet appreciated that *spoT1* has two mutant domains, one affects synthesis (R140C) and the other alters hydrolysis (a QD insertion between residues 82 and 83). Together, they resulted in greatly prolonged cellular ppGpp decay rates that were interpreted solely in terms of a hydrolysis defect. It was also noticed that pppGpp failed to accumulate if a *spoT1 relA+* strain was starved for amino acids or glucose while a wt (*spoT+ relA+*) starved for amino acids, but not glucose, accumulated pppGpp and ppGpp. Thus, the wild type *spoT* gene controlled “spottiness,” which gave the *spoT* gene its name (Laffler and Gallant, 1974; Stamminger and Lazzarini, 1974). The presence of regulated pppGpp during glucose starvation has been observed earlier (Gentry and Cashel, 1996). Our observations here during the diauxic shift (**Figure 3**), entails a glucose exhaustion modulated by the accompanying utilization of a secondary sugar. It is still true that currently almost nothing is known as to how carbon source limitation provokes ppGpp accumulation by SpoT and whether this occurs by regulating hydrolysis, synthesis or both. Nevertheless, compelling evidence is provided by transcriptomics that changes of gene expression do occur during glucose–lactose diauxie and that they are correlated with the presence of ppGpp (Traxler et al., 2006).

Here, relatively simple approaches with a well-studied system reveal a high level of complexity. Comparisons of diauxic lag time differences among a set of three strains: wt with both synthetases and two mutant strains constructed with alleles that provide either RelA or SpoT as the sole source of ppGpp synthesis. All three have the same wt hydrolysis domain encoding apparently similar activities (Supplementary Figure S2). Estimates of the diauxic lag times of this strain set under various conditions seem to reveal a somewhat more fundamental understanding of diauxie. While these measurements focus on the contributions of the *spoT* and *relA* synthetases to the diauxic lag times needed to adapt to changing carbon sources, the mechanistic interpretation suggests a general mechanism by which ppGpp might accomplish this feat regardless of the synthetic source of ppGpp. Moreover, similar results are obtained with high or low ppGpp, showing that diauxic lag time are controlled by a balanced synthesis vs. hydrolysis activity. The notion that more ppGpp is better, resulting in faster adaptation seems uncertain at best.

The higher amounts of ppGpp detected in the SpoT E319Q mutant could be explained due some deficiency in the activation of the SpoT hydrolase, although its hydrolysis activity is intact (Supplementary Figure S2C). This possibility arises from the apparent conformational changes suggested during the binding of ACP to SpoT, switching the balance between synthesis and hydrolysis activities (Battesti and Bouveret, 2006). To keep a proper balance, similar conformational changes may occur during the diauxic shift that could be partially impaired by the E319Q mutation. Analogous conformational and catalytic activity changes are also found with the catalytic domains of the bifunctional Rel*Seq* RSH protein (Hogg et al., 2004).

During a diauxic shift when MG1655 exhausts glucose and undergoes a lag time of up to 40 min to adjust gene expression patterns to adapt to lactose accompanied by the synthesis of new enzymes and transporters while consuming energy and amino acids. The effects of ppGpp over the diauxic growth shift seem to minimally involve classical regulators (**Figure 2**), such as CRP or Mlc, although cAMP-CRP effects indirectly have strong regulatory effects on many pathways affected by diauxie (Supplementary Figure S1). The proposed mechanism for ppGpp involvement occurs at the level of altered abundance of acetyl-phosphate. Growth on glucose is accompanied by secretion of acetate. As shown in Enjalbert et al. (2017), acetate can reenter cells while growing on glucose mainly by the SatP transporter (**Figure 4**). Although ATP is required in the first step of the reaction that converts Ac-P into acetyl-CoA, it will enter the TCA cycle and produce more energy (Enjalbert et al., 2017). RT-PCR measurements reveal the levels of both *ackA* and *pta* mRNA are lower than wt in a *ΔrelA* mutant and drop in parallel during the diauxic lag and thereafter during outgrowth (Supplementary Figure S3). We propose the ppGpp activating mechanism could involve stimulation of two promoters upstream of the *ackA-pta* operon because both because both contain A-T rich discriminator sequences (Supplementary Figure S3). During acetate utilization, the Ac-P levels in this pathway could be increased by a more active AckA enzyme perhaps coupled with a less active Pta activity. The mechanisms controlling Ac-P accumulation remain unknown, although the correlation between basal levels of ppGpp and Ac-P found here suggest ppGpp can be involved (**Figure 5C**). Once formed, Ac-P could acetylate a variety of different proteins during diauxic shifts (**Figure 6**) that are related to the glucose metabolism (Kuhn et al., 2014; Venkat et al., 2017). Another possibility arises from a recent study (Davis et al., 2018) that show that acetylation of CRP can also control its activity. Although ppGpp is not controlling the diauxic shift through changes in CRP expression, it might control it by changing its activity through acetylation.

Amino acids as well as succinate eliminate the effect of the lack of the ppGpp synthetases (**Figure 1D**), but we think probably through different mechanisms. Although succinate can be used to synthesize some amino acids and it can be formed from other amino acids, it seems that both effects are not directly related. Adding the full set of amino acids probably does eliminate the activation of RelA by restoring tRNA charging while succinate blocks acetate uptake (**Figure 4**). However, succinate also leads to depletion of ppGpp levels when SpoT synthetase is the only source of ppGpp (**Figure 3**), showing a possible decrease on synthesis or increase on hydrolysis activity.

Studies of co-utilized carbon sources (Hermsen et al., 2015) allow quantitatively predictions of growth rates of cultures using both sugars based on the growth on each sugar alone. If glucose and succinate were co-utilized as carbon source, few effects are predicted. Instead we observe an increase of the growth rate from 0.47 to 0.66 duplication/hour when succinate is added. That could implicate involvement of succinate in the synthesis of some amino acids.

As previously mentioned, most of the studies showing acetate overflow (Wolfe, 2005; Sá-Pessoa et al., 2013; Enjalbert et al., 2017) or effects of Ac-P over protein acetylation (Kuhn et al., 2014; Schilling et al., 2015), were performed in media containing high glucose (1–2%). In this study, we can show protein acetylation occurs even during the diauxic shift under more common laboratory conditions for bacterial growth.

This work leads to the suggestion that a role for ppGpp adds to the many complexities already known to accompany regulatory adjustment of diauxic shifts. As just mentioned the details of how SpoT senses glucose starvation remain elusive, not to mention the transition into utilization of alternative carbon sources. Further studies are required to identify and define these possible mechanisms. For example, the uptake of acetate would alter the abundance of acetyl-CoA, which in turn it may alter lipid synthesis, acylation of ACP, which can then alter the ppGpp balance of SpoT synthesis and hydrolysis by binding of to the SpoT TGS domain.

## Author Contributions

LF-C and MC conceived and designed the study, performed experiments, contributed to the data analysis, and drafted and revised the manuscript. Both authors have read and approved the final version of this manuscript.

## Conflict of Interest Statement

The authors declare that the research was conducted in the absence of any commercial or financial relationships that could be construed as a potential conflict of interest.
