# Supplementary material for: Contributions of SpoT Hydrolase, SpoT Synthetase, and RelA Synthetase to Carbon Source Diauxic Growth Transitions in Escherichia coli
Source: Front Microbiol. 2018 Aug 3;9:1802. doi: 10.3389/fmicb.2018.01802 (PMC6085430; doi:10.3389/fmicb.2018.01802)
Supplement: Supplementary file 1 [file Image_1.pdf]

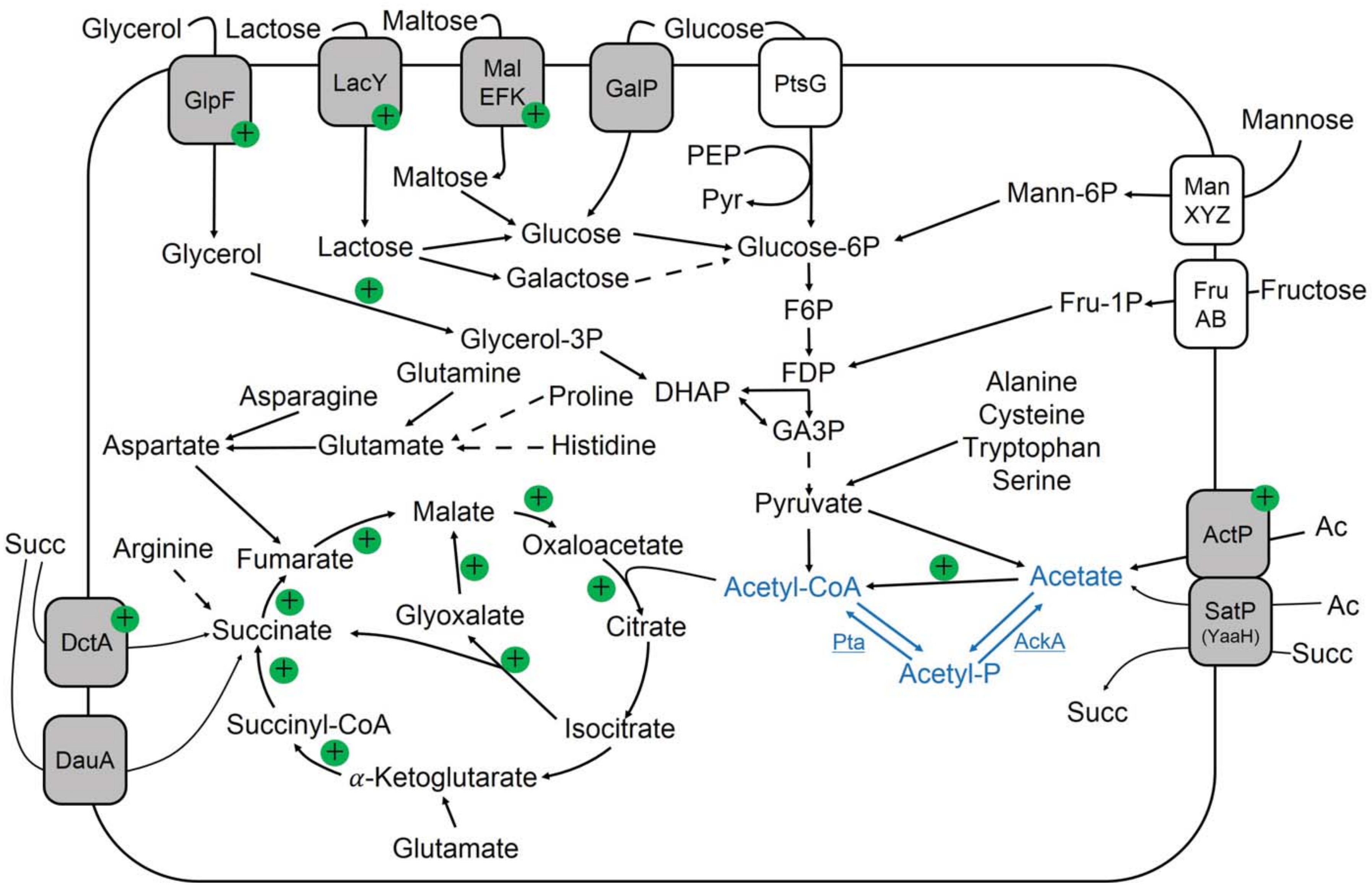

Figure S1. Carbohydrate metabolism and transport. Central metabolism including transport and metabolic pathways of the sugars used in this study. In blue is shown the synthesis and degradation pathway of Acetyl-Phosphate. ⊕ genes that require CRP for its expression. PTS transporters are indicated as in Figure 1: PTS in white and Non-PTS in gray. (McFall and Newman, 1996; Neidhardt and Curtis, 1996; Wang et al., 1998; Gosset et al., 2004; Traxler et al., 2006; Sá-Pessoa et al., 2013)

References

Gosset, G., Zhang, Z., Nayyar, S., Cuevas, W. A., and Saier, M. H. (2004). Transcriptome Analysis of Crp-Dependent Catabolite Control of Gene Expression in Escherichia coli. *J. Bacteriol.* 186, 3516–3524. doi:10.1128/JB.186.11.3516-3524.2004.

McFall, E., and Newman, E. B. (1996). "Amino Acids as Carbon Sources," in *Escherichia coli and Salmonella: cellular and molecular biology*, 358–379.

Neidhardt, F. C., and Curtis, R. (1996). *Escherichia coli and salmonella : cellular and molecular biology*. Washington, D.C. : ASM Press.

Sá-Pessoa, J., Paiva, S., Ribas, D., Silva, I. J., Viegas, S. C., Arraiano, C. M., et al. (2013). SATP (YaaH), a succinate-acetate transporter protein in Escherichia coli. *Biochem. J.* 454, 585–95. doi:10.1042/BJ20130412.

Traxler, M. F., Chang, D.-E., and Conway, T. (2006). Guanosine 3',5'-bispyrophosphate coordinates global gene expression during glucose-lactose diauxie in Escherichia coli. *Proc. Natl. Acad. Sci. U. S. A.* 103, 2374–9. doi:10.1073/pnas.0510995103.

Wang, Y. P., Kolb, A., Buck, M., Wen, J., O'Gara, F., and Buc, H. (1998). CRP interacts with promoter-bound sigma54 RNA polymerase and blocks transcriptional activation of the dctA promoter. *EMBO J.* 17, 786–96. doi:10.1093/emboj/17.3.786.
