# Supplementary material for: Contributions of SpoT Hydrolase, SpoT Synthetase, and RelA Synthetase to Carbon Source Diauxic Growth Transitions in Escherichia coli
Source: Front Microbiol. 2018 Aug 3;9:1802. doi: 10.3389/fmicb.2018.01802 (PMC6085430; doi:10.3389/fmicb.2018.01802)
Supplement: Supplementary file 2 [file Image_2.PDF]

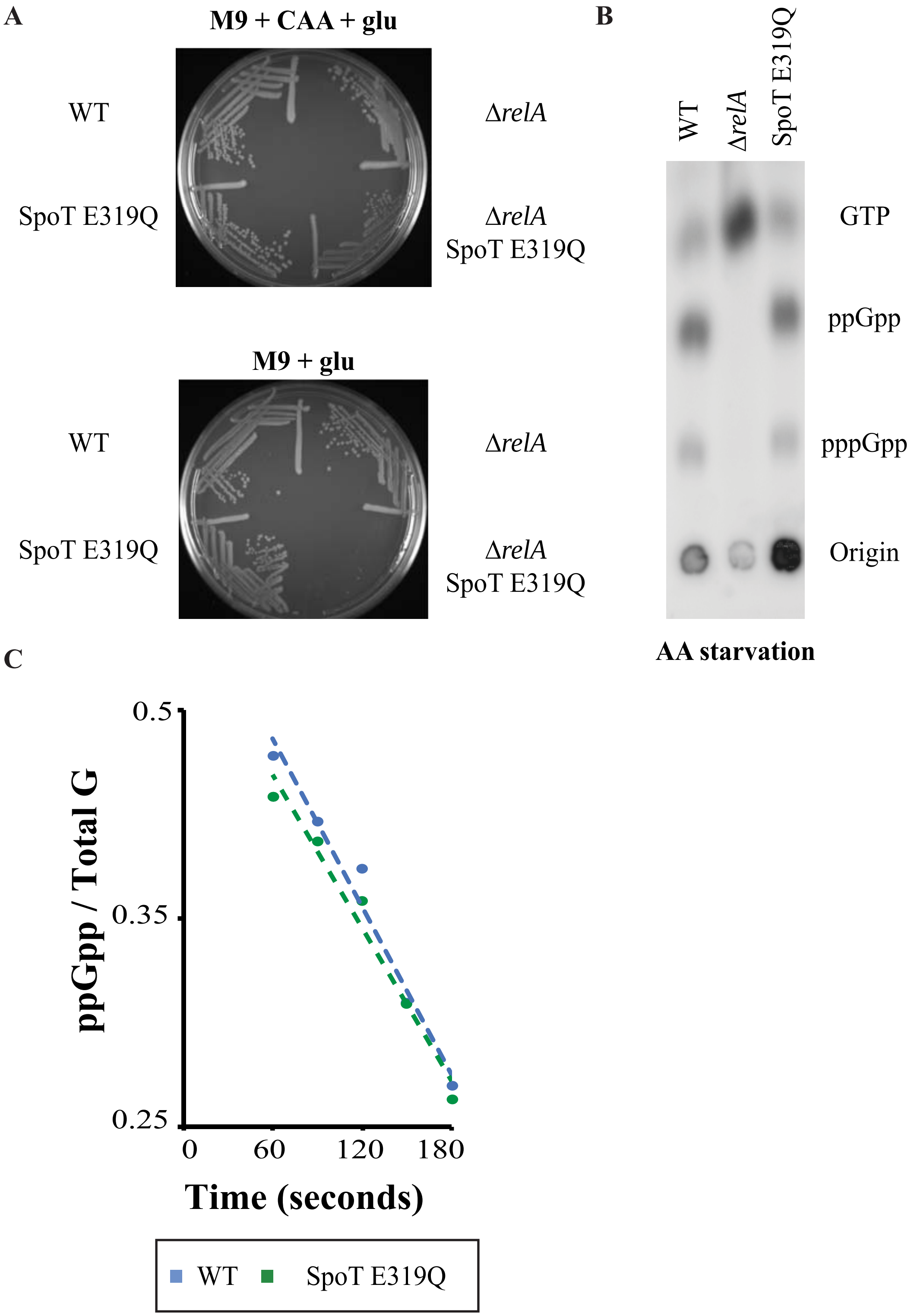

Figure S2. Effects of SpoT E319Q on ppGpp synthesis and degradation. **(A)** Growth of WT, Δ*relA*, SpoT E319Q and Δ*relA* SpoT E319Q strains in M9 + glucose with and without 0.4% CAA. The Δ*relA* SpoT E319Q double mutant does not grow in M9 + glucose, indicating the complete absence of ppGpp. **(B)** ppGpp levels on strains WT, Δ*relA* and SpoT E319Q induced by SHX-inhibition of serine aminoacylation. **(C)** ppGpp degradation rates are similar after induction by 1 mg/ml SHX treated WT and SpoT E319Q strains then reversed by adding 200 µg/ml Cm.
