# Supplementary material for: Contributions of SpoT Hydrolase, SpoT Synthetase, and RelA Synthetase to Carbon Source Diauxic Growth Transitions in Escherichia coli
Source: Front Microbiol. 2018 Aug 3;9:1802. doi: 10.3389/fmicb.2018.01802 (PMC6085430; doi:10.3389/fmicb.2018.01802)
Supplement: Supplementary file 3 [file Image_3.pdf]

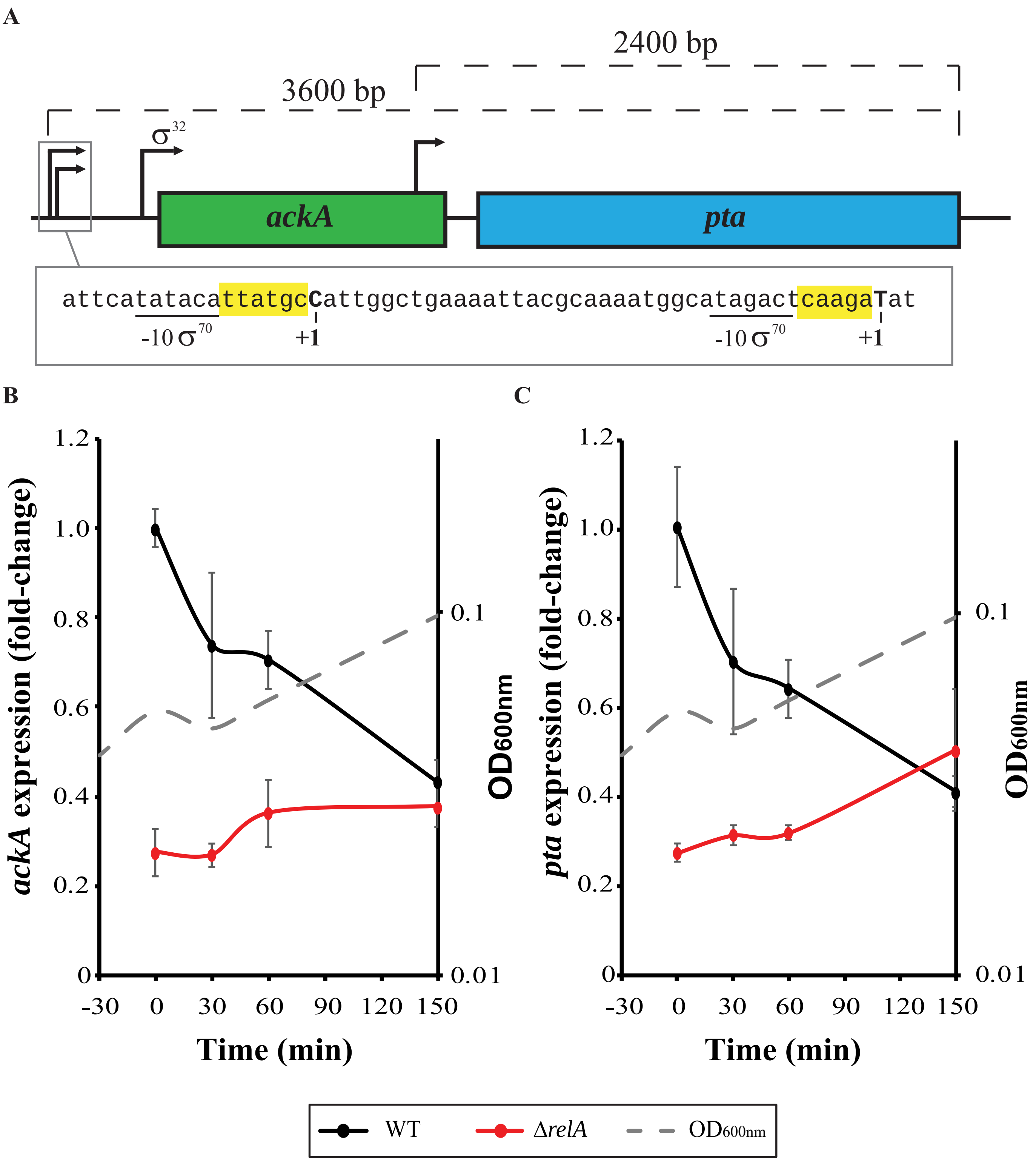


**Figure S3.** Expression levels of *ackA* and *pta* during the diauxic shift in WT and Δ*relA* strains. **(A)** The operon *ackA*-*pta* is depicted with its predicted promoters, based on transcript size (Kakuda et al., 1994), TSS position from RNAseq data (Salgado et al., 2013) and prediction of -10 boxes (Münch et al., 2005). The σ32-dependent promoter (Gama-Castro et al., 2007)is also shown. Discriminator areas are highlighted in yellow. **(B)** and **(C)** show the expression levels of *ackA* and *pta* (respectively) during the diauxic shift in presence or absence of RelA. Average and standard deviation from two independent cultures in triplicate are presented.

## References

Gama-Castro, S., Jimenez-Jacinto, V., Peralta-Gil, M., Santos-Zavaleta, A., Penaloza-Spinola, M. I., Contreras-Moreira, B., et al. (2007). RegulonDB (version 6.0): gene regulation model of Escherichia coli K-12 beyond transcription, active (experimental) annotated promoters and Textpresso navigation. *Nucleic Acids Res.* 36, D120–D124. doi:10.1093/nar/gkm994.

Kakuda, H., Hosono, K., Shiroishi, K., and Ichihara, S. (1994). Identification and characterization of the ackA (acetate kinase A)-pta (phosphotransacetylase) operon and complementation analysis of acetate utilization by an ackA-pta deletion mutant of Escherichia coli. *J. Biochem.* 116, 916–22.

Münch, R., Hiller, K., Grote, A., Scheer, M., Klein, J., Schobert, M., et al. (2005). Virtual Footprint and PRODORIC: an integrative framework for regulon prediction in prokaryotes. *Bioinformatics* 21, 4187–9. doi:10.1093/bioinformatics/bti635.

Salgado, H., Peralta-Gil, M., Gama-Castro, S., Santos-Zavaleta, A., Muñiz-Rascado, L., García-Sotelo, J. S., et al. (2013). RegulonDB v8.0: omics data sets, evolutionary conservation, regulatory phrases, cross-validated gold standards and more. *Nucleic Acids Res.* 41, D203–D213. doi:10.1093/nar/gks1201.
