# Supplementary material for: Contributions of SpoT Hydrolase, SpoT Synthetase, and RelA Synthetase to Carbon Source Diauxic Growth Transitions in Escherichia coli
Source: Front Microbiol. 2018 Aug 3;9:1802. doi: 10.3389/fmicb.2018.01802 (PMC6085430; doi:10.3389/fmicb.2018.01802)
Supplement: Supplementary file 4 [file Image_4.PDF]

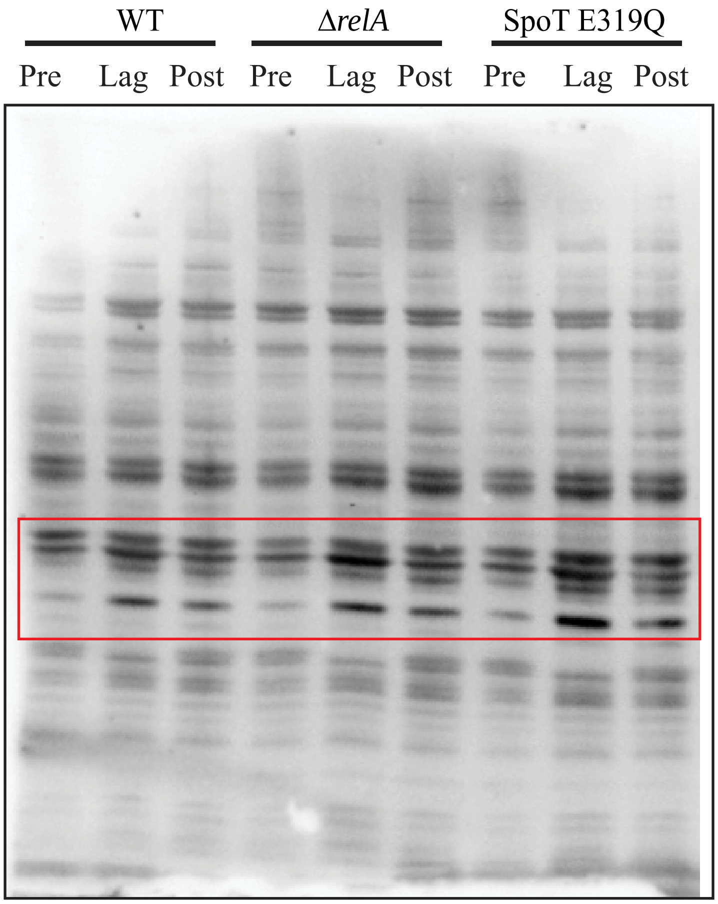

Figure S4. Western blots with monoclonal antibody against acetylated lysine at different times during diauxic shifts for the strain set: wt, Δ*relA* and SpoT E319Q. Full Western Blot from figure 6. Red box correponds to the tracing shown in figure 6B.
