# Supplementary material for: Contributions of SpoT Hydrolase, SpoT Synthetase, and RelA Synthetase to Carbon Source Diauxic Growth Transitions in Escherichia coli
Source: Front Microbiol. 2018 Aug 3;9:1802. doi: 10.3389/fmicb.2018.01802 (PMC6085430; doi:10.3389/fmicb.2018.01802)
Supplement: Supplementary file 5 [file Table_1.pdf]

Table S1: Bacterial strains and plasmids used in this study

Strains

**Name**	**Properties**	**Origin**
MG1655	F-, *ilvG*, *rph*1	(Jin and Gross, 1988)
CF18005	MG1655 Δ*relA*256 (CF12510)	(Potrykus et al., 2011)
CF18011	MG1655 SpoT E319Q zib563::tn10 (CF15523)	(Harinarayanan et al., 2008)
LFC1204	MG1655 *crp5*::Cm pHA7	This study
LFC1206	CF18005 *crp5*::Cm pHA7	This study
LFC1208	CF18011 *crp5::*Cm pHA7	This study
LFC1209	MG1655 *fruR*::Km	This study
LFC1211	CF18005 *fruR*::Km	This study
LFC1213	CF18011 *fruR*::Km	This study
LFC1210	MG1655 *mlc*::Km	This study
LFC1212	CF18005 *mlc*::Km	This study
LFC1214	CF18011 *mlc*::Km	This study
CF6270	MG1655 Δ*cyaA ilvC*::Tn10 Tc^R	Cashel Lab
CF6271	MG1655 *crp5*::Cm	Cashel Lab
LFC1391	MG1655 *dauA*::Km	This study
LFC1392	CF18005 *dauA*::Km	This study
LFC1393	CF18011 *dauA*::Km	This study
LFC1394	MG1655 *dctA*::Km	This study
LFC1395	CF18005 *dctA*::Km	This study
LFC1396	CF18011 *dctA*::Km	This study
LFC1397	MG1655 *yaaH*::Km (*satP*)	This study
LFC1398	CF18005 *yaaH*::Km (*satP*)	This study
LFC1399	CF18011 *yaaH*::Km (*satP*)	This study
LFC1304	MG1655 *ackA*::Km	This study
LFC1306	CF18005 *ackA*::Km	This study
LFC1308	CF18011 *ackA*::Km	This study
LFC1303	MG1655 *pta*::Km	This study
CF18006	MG1655 Δ*relA* SpoT R39A zib563::tn10 (CF17956)	(Harinarayanan et al., 2008)
CF18565	MG1655 Δ*ackA* Δ*pta* zej223::tn10	This study

Plasmids

**Name**	**Properties**	**Origin**
pHA7	Ap^R, pBR322-CRP (without *crp* promoter)	(Aiba et al., 1982)

References

Aiba, H., Fujimoto, S., and Ozaki, N. (1982). Molecular cloning and nucleotide sequencing of the gene for E. coli cAMP receptor protein. *Nucleic Acids Res.* 10, 1345–61.

Harinarayanan, R., Murphy, H., and Cashel, M. (2008). Synthetic growth phenotypes of Escherichia coli lacking ppGpp and transketolase A (tktA) are due to ppGpp-mediated transcriptional regulation of tktB. *Mol. Microbiol.* 69, 882–94. doi:10.1111/j.1365-2958.2008.06317.x.

Jin, D. J., and Gross, C. A. (1988). Mapping and sequencing of mutations in the Escherichia colirpoB gene that lead to rifampicin resistance. *J. Mol. Biol.* 202, 45–58. doi:10.1016/0022-2836(88)90517-7.

Potrykus, K., Murphy, H., Philippe, N., and Cashel, M. (2011). ppGpp is the major source of growth rate control in E. coli. *Environ. Microbiol.* 13, 563–75. doi:10.1111/j.1462-2920.2010.02357.x.
