# Supplementary material for: Contributions of SpoT Hydrolase, SpoT Synthetase, and RelA Synthetase to Carbon Source Diauxic Growth Transitions in Escherichia coli
Source: Front Microbiol. 2018 Aug 3;9:1802. doi: 10.3389/fmicb.2018.01802 (PMC6085430; doi:10.3389/fmicb.2018.01802)
Supplement: Supplementary file 6 [file Table_2.PDF]

Table S2: List of primers used in this report.

Name	Sequence (from 5' → 3')
parC1	ATGGATCAGGTGATGAACCA
parC2	AGAGGATTTCCAGCAGGTTT
pta1	AACTGAACGCACCGGTTGAT
pta2	GAAGAGTCGTCGAAAATCTC
ackA1	CAAACTGCTGACCAAAGAGT
ackA2	GCGGTAGTTGTCTTCAACAT
